# DEVELOPMENT AND ACCEPTABILITY OF A MOBILE APP FOR MONITORING EVERYDAY FUNCTIONAL ABILITIES

**DOI:** 10.1093/geroni/igad104.2203

**Published:** 2023-12-21

**Authors:** Ava Mcvey, Kalvry Cooper, Abigail Stephan, Tatum Steele, Lesley Ross, Christine Phillips

**Affiliations:** Clemson University, Clemson, South Carolina, United States; Clemson University, Clemson, South Carolina, United States; Clemson University, Clemson, South Carolina, United States; Clemson University, Clemson, South Carolina, United States; Clemson University, Clemson, South Carolina, United States; Clemson University, Clemson, South Carolina, United States

## Abstract

Subtle changes in instrumental activities of daily living (IADL), also termed everyday functional abilities, may be early markers of dementia. However, there are no widely available tools for tracking IADL function, and it is unclear whether such a tool would be used by older adults. The present study investigates older adults’ adherence to a novel mobile app designed to conveniently and cost-effectively monitor IADL function. We also examine participants’ acceptability of the app, including their overall satisfaction, perceived ease of use, benefits and limitations, and suggestions for improvement. Community-dwelling older adults (n=24; Mage=71.63 years) were randomly assigned three different simulated IADL tasks, to complete at specified times, each day for two weeks. Adherence was assessed using app usage data. App perceptions were assessed via survey and individual interview at the end of the two-week study period. Results indicate high adherence, with an average total task completion of 94.2%. Results from follow-up interviews revealed the app was generally perceived as “easy to use” and the assigned tasks could be completed quickly. Participants expressed frustration over app glitches that prevented them from successfully completing tasks and noted the assigned tasks and associated steps to complete were too simplified to be realistic. High adherence rates and general acceptance indicate the app’s potential usefulness. Further research should assess long-term effectiveness and user experience, especially among a more diverse sample. If reliable and valid, such apps could enable early dementia detection, but only if their use is feasible and accepted.

